# 3D-Printed Antibiotic Beads with Controlled Microarchitectures for Customizable Elution Profiles: An In Vitro Study

**DOI:** 10.1007/s43465-026-01719-z

**Published:** 2026-02-25

**Authors:** Vivek K. Bilolikar, Duncan Van Vannest, Clark Chen, Solomon P. Samuel, John A. Handal, James S. Raphael

**Affiliations:** 1 Department of Orthopaedics, Jefferson Einstein Philadelphia Hospital – Jefferson Health, 5501 Old York Rd, WCB4, Philadelphia, PA 19141 USA; 2 Sidney Kimmel Medical College, Thomas Jefferson University, 1020 Walnut St, Philadelphia, PA 19107 USA

**Keywords:** 3D printing, Bone cement, Antibiotics, Porogen, CAD, Infection management, Customized drug delivery, Osteomyelitis, Prosthetic joint infection

## Abstract

**Introduction:**

Polymethylmethacrylate (PMMA) antibiotic beads are widely used in orthopedic surgery for localized infection control, but inconsistent drug elution can limit efficacy. Three-dimensional (3D) printing offers a way to create implants with controlled architectures, enabling more predictable and customizable drug release. This study explores the use of internal structural variation to influence the elution profiles of vancomycin and tetracycline and assess their antibacterial properties.

**Methods:**

Cylindrical specimens were 3D printed using a commercial resin mixed with 2% vancomycin or tetracycline. To evaluate the effects of composition and structure, additives such as sodium chloride (NaCl) and polyvinylpyrrolidone (PVP) were included, and four internal architectures—solid, hollow, large honeycomb, and small honeycomb—were tested. Elution was assessed over 7 days, and antibacterial activity was tested against *S. epidermidis* and *E. coli* using agar diffusion.

**Results:**

Tetracycline exhibited greater cumulative release than vancomycin, likely due to solubility and molecular weight differences. Honeycomb structures, especially the smaller variant, produced the most sustained and consistent elution profiles. NaCl decreased vancomycin but increased tetracycline release, while PVP enhanced release for both. All formulations showed antibacterial efficacy.

**Discussion:**

3D printing enables the design of antibiotic-eluting implants with tailored release profiles. The honeycomb architecture is especially promising for sustained delivery. Further in vivo studies are needed to confirm clinical relevance and optimize formulations for orthopedic use.

## Introduction

Antibiotic-loaded polymethyl methacrylate (PMMA) has become an indispensable tool for delivering high concentrations of antibiotics directly to infection sites, particularly in cases of osteomyelitis, post-surgical infections, and implant-related infections [1–3]. Traditional PMMA-based local antibiotic delivery systems, including antibiotic-loaded bone cement, beads, and spacers, are designed to achieve an initial high “burst release” of antibiotics, followed by a sustained release over several weeks. However, during the prolonged release phase, antibiotic concentrations may fall below the minimum inhibitory concentration (MIC), compromising therapeutic effectiveness and potentially allowing bacterial regrowth [4].

A critical limitation of PMMA-based antibiotic delivery systems lies in their preparation process prior to implantation. PMMA is typically mixed manually in the operating room, where it is molded into beads, spacers, or applied directly into bone cavities. This manual process varies by operator, often resulting in inconsistent antibiotic distribution within the cement and uneven drug elution. Furthermore, the polymerization of bone cement traps a substantial portion of the antibiotic within the PMMA matrix, limiting its release. Efforts to improve drug elution through the incorporation of porogens and soluble fillers have achieved only modest success, underscoring the need for more effective solutions [5–7]. Such inconsistencies in drug release hinder infection control, as subtherapeutic antibiotic concentrations may fail to suppress infection or prevent bacterial regrowth.

Advancements in 3D-printing technology present a promising alternative to traditional bone cement preparation methods by enabling the design of drug-eluting bone cements with customizable shapes and internal architectures. This technology offers precise control over features such as bead or spacer size, internal porosity, and surface roughness, significantly enhancing drug delivery performance. When combined with strategies such as the incorporation of porogens, 3D printing can optimize elution profiles, enabling more consistent, prolonged, or even controlled, timed drug release. These innovations have the potential to improve local drug delivery, aligning antibiotic availability with the dynamic requirements of infection management and specific treatment phases.

Currently, no off-the-shelf 3D-printable PMMA-based products are available, limiting the direct application of 3D printing to PMMA systems. In orthopedics, alternatives like bisphenol-A-glycidyl methacrylate (Bis-GMA)-based resins, which are light-curable and 3D printable [9, 10], have been successfully used for bone augmentation and fracture repair [8]. However, these formulations are not readily commercially available as 3D-printable resins. Inspired by the versatility of 3D-printable resin systems, we aimed to investigate a novel approach: leveraging customized designs and tailored internal architectures to enhance antibiotic elution profiles and antibacterial activity from bone cement structures. For this proof-of-concept study, we utilized a commercially available light-curable acrylate resin—not a Bis-GMA formulation—to evaluate whether such tailored internal geometries could optimize drug release.

Acrylate-based resins have a long history of applications in medicine and dentistry. They are used for fabricating high-precision temporary dental implants and orthopedic devices like custom surgical guides, leveraging their ability to create complex geometries. The production process creates a high cross-link density that ensures the necessary stiffness and strength for these short-term, load-bearing applications.

This study investigates the potential of 3D printing to optimize local antibiotic delivery by addressing key limitations in traditional systems. Specifically, we evaluated three factors: the elution profiles of antibiotics (vancomycin and tetracycline) from 3D-printed resins, the effects of internal structural modifications (e.g., honeycomb designs with varying pore sizes), and the impact of additives such as sodium chloride or polyvinylpyrrolidone (PVP) on drug release [5, 6, 11]. By examining these parameters, this work establishes a foundation for developing 3D-printable bone cement systems capable of overcoming current drug elution challenges, enabling more personalized and effective infection management in orthopedic care.

## Materials and Methods

### Preparation of Tetracycline/Vancomycin-Loaded Resin Compositions

Three different antibiotic-loaded resin formulations were prepared to evaluate the effects of additives and internal structure on antibiotic elution profiles. Tetracycline and vancomycin were selected due to their differing molecular weights (vancomycin: 1,449.3 g/mol; tetracycline: 444.4 g/mol).

1. Antibiotic-only resin
2. Fifty milliliters of Anycubic Clear resin was mixed with 500 mg of antibiotic. The antibiotic was pre-crushed into a fine powder using a pestle and mortar to ensure uniform mixing.
3. Antibiotic with NaCl resin
4. Fifty milliliters of Anycubic Clear resin was mixed with 500 mg of antibiotic and 500 mg of sodium chloride (NaCl). Both the antibiotic and NaCl were pre-crushed into fine powders before incorporation into the resin.
5. Antibiotic with polyvinylpyrrolidone (PVP) resin
6. Fifty milliliters of Anycubic Clear resin was mixed with 500 mg of antibiotic and 500 mg of polyvinylpyrrolidone (PVP; average molecular weight: 360,000 g/mol). The antibiotic and PVP were pre-crushed into fine powders to ensure homogeneity during mixing.

All mixtures were mixed using a planetary centrifugal mixer (Thinky U.S.A., Inc, Laguna Hills, CA) to achieve uniform consistency, ensuring even distribution of the antibiotics and additives within the resin matrix. The prepared formulations were subsequently used for 3D printing and analysis.

### Specimen Fabrication and Design

Cylindrical specimens were fabricated using a stereolithography 3D printer (Anycubic Photon printer, Hongkong Anycubic Technology CO., LTD) to evaluate how internal architecture affects antibiotic release. Four distinct internal structures were designed using Microsoft 3D Builder software: a solid cylinder, a cylindrical tube, a large honeycomb, and a small honeycomb. For printing, a prepared antibiotic-resin mixture was poured into the printer’s tray. The specimens, measuring 12 mm in height and 12 mm in diameter, were printed with a layer height of 0.075 mm and a 2.2-s exposure time. After printing, each specimen was simply wiped with a paper towel to remove uncured resin.

### Design of Internal Structures

Four distinct internal structures were designed using Microsoft 3D Builder software and 3D printed to evaluate variations in drug release: solid cylinder, cylindrical tube, large honeycomb, and small honeycomb. We acknowledge that the internal microarchitectures tested here are a preliminary exploration, and more optimized designs likely exist.

- The solid cylinders featured fully filled cores, while the cylindrical tubes had a wall thickness of 3 mm.
- The honeycomb structures varied in internal surface area and void volume. Large honeycomb cells measured approximately 4 mm per side, while smaller honeycomb cells measured 2 mm per side.

These designs were selected to investigate how differences in internal geometry influence drug elution profiles and mechanical stability. It was hypothesized that structures with increased internal surface area, such as honeycomb designs, might facilitate more consistent or prolonged drug release. Hollow cylinders were used in a comparative analysis to evaluate differences in elution between vancomycin- and tetracycline-loaded specimens.

### Elution Profile Assessment

Elution profiles were assessed in vitro by immersing six antibiotic-loaded specimens in plastic vials containing 25 mL of saline (elution medium). The vials were incubated at 37 °C, and antibiotic elution was measured at various time points over 120 h. Measurements were performed in triplicate using a microplate reader (Spectramax 190, Molecular Devices, CA) at wavelengths of 280 nm for vancomycin and 357 nm for tetracycline. The elution medium was replaced after each measurement. The total amount of antibiotic eluted was calculated at the end of the 120-h period.

### Antibacterial Assay

An agar diffusion assay was conducted to confirm that the antibiotics retained their antibacterial properties after the 3D-printing process. Thin cylindrical discs containing the respective antibiotic, without any porogen, were 3D printed for testing.

- Bacterial strains: *Escherichia coli* was used for tetracycline, and *Staphylococcus epidermidis* was used for vancomycin.
- Agar plate preparation: agar plates were inoculated with the respective bacterial suspension to ensure uniform distribution.

The 3D-printed specimens were placed on the inoculated agar plates and incubated at 37 °C for 24 h. Control groups included resin discs without antibiotics and standard antibiotic discs. The presence of inhibition zones around the 3D-printed specimens confirmed that the antibiotics retained their antibacterial activity post-printing.

## Results

The antibiotics and porogens were successfully incorporated into the commercially available resin using a centrifugal planetary mixer without significant difficulty. However, the high viscosity of the resin mixture necessitated the use of larger 3D printers capable of handling high-viscosity materials. The 3D-printed specimens hardened fully upon completion and exhibited excellent structural integrity, with no chipping or damage observed during removal from the print platform.

The elution experiments were conducted using 3D-printed cylindrical specimens with increasing surface areas achieved through different internal structural designs. The structures included solid cylinders, cylindrical tubes, cylinders with large internal channels, and cylinders with small internal channels.

### Tetracycline Elution Results

The results demonstrated that tetracycline elution was highest in specimens with smaller internal channels under standard conditions. However, when polyvinylpyrrolidone (PVP) was used as a porogen, the highest elution occurred in specimens with large internal channels.

**Fig. 1:**
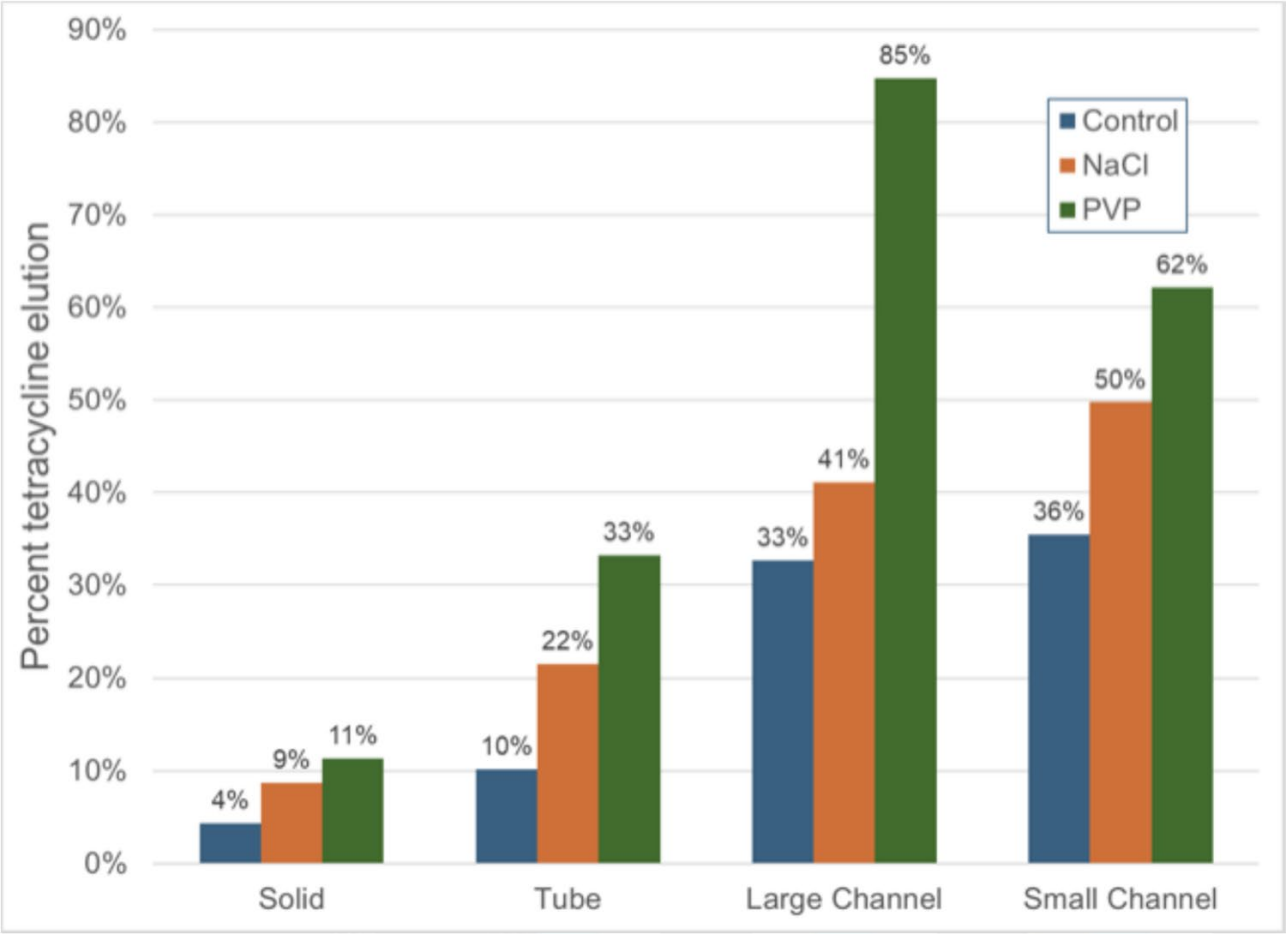
Percent of tetracycline released as stratified by bead microarchitecture and porogen

The addition of sodium chloride (NaCl) and PVP significantly enhanced tetracycline release across all specimen designs (Table 1). The elution percentages for different designs are summarized as follows and illustrated in Fig. 1:

- Solid specimens: Tetracycline elution increased from 4% in the control (no porogen) to 9% with NaCl and 11% with PVP.
- Tube designs: Elution rose from 10% in the control to 22% with NaCl and 33% with PVP.
- Specimens with large internal channels: The most pronounced effect was observed, with tetracycline release increasing from 33% in the control to 41% with NaCl and 85% with PVP.
- Specimens with small internal channels: Elution increased from 36% in the control to 50% with NaCl and 62% with PVP.

**Table 1:** The total amount (mg) of tetracycline eluted within 120 h from 6 specimens each

	Tetracycline only	Tetracycline + NaCl	Tetracycline + PVP
Solid	8	13	16
Tube	13	25	32
Large honeycomb	17	20	41
Small honeycomb	18	24	31

Among the porogens tested, PVP consistently resulted in the greatest enhancement of drug release, particularly in large-channel specimens. This finding suggests that the combination of a high molecular weight porogen, such as PVP, with increased surface area can significantly improve tetracycline elution. These results underscore the importance of tailoring internal structures and porogen selection to optimize drug release profiles.

### Vancomycin Elution Results

**Fig. 2:**
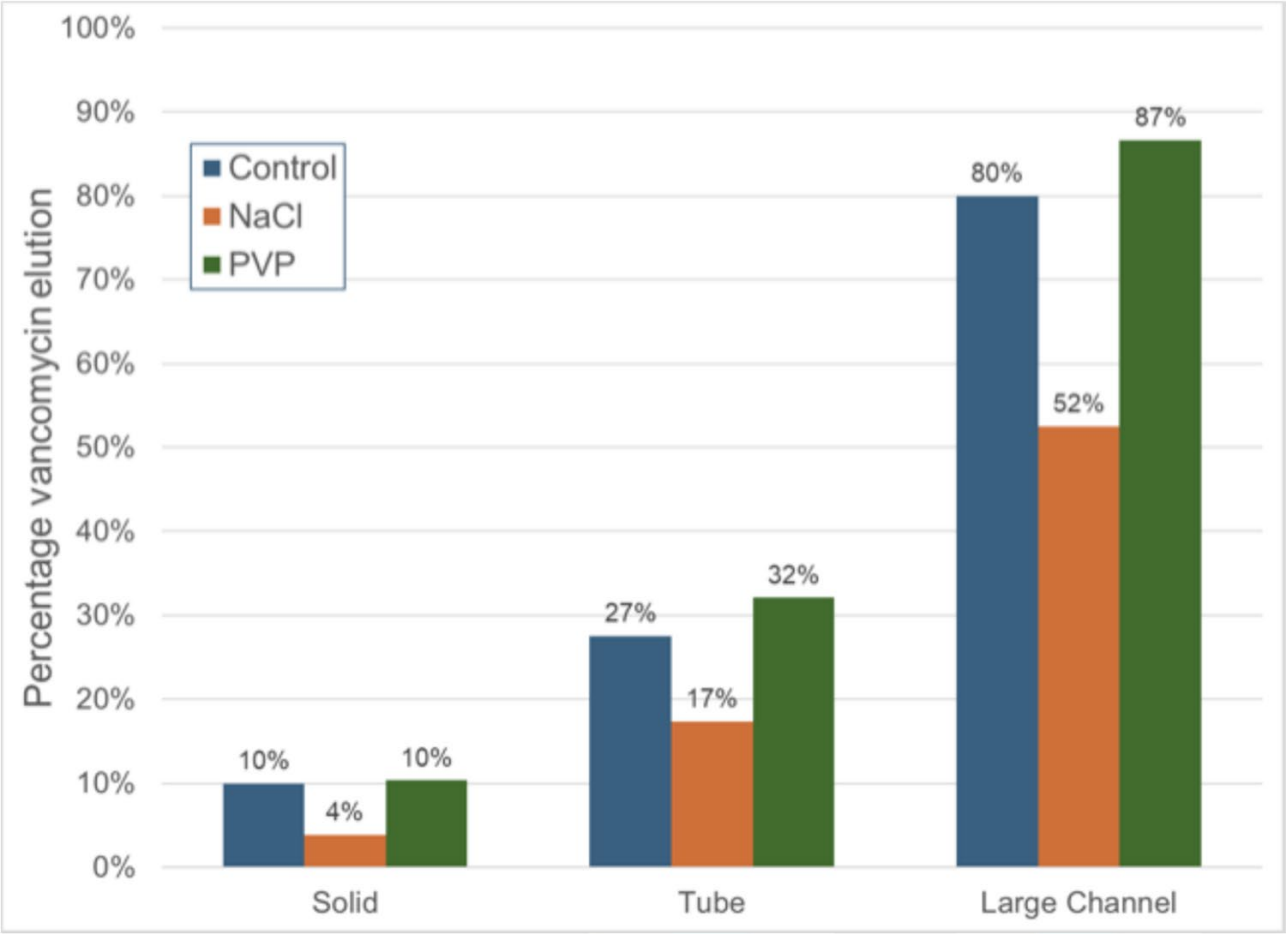
Percent of vancomycin released as stratified by bead microarchitecture and porogen

Vancomycin elution was highest in hollow specimens compared to solid specimens, with this difference being further amplified by the addition of polyvinylpyrrolidone (PVP) as a porogen (see Fig. 2).

The addition of sodium chloride (NaCl) consistently reduced vancomycin elution across all structural types (see Table 2). Specifically, the elution percentages for vancomycin were as follows:

- Solid specimens: Elution was 10% in the control, 4% with NaCl, and 10% with PVP.
- Tube structures: Elution increased from 27% in the control to 17% with NaCl and 32% with PVP.
- Large channel honeycomb specimens: The most significant effect was observed, with elution rising from 80% in the control to 52% with NaCl and 87% with PVP.

**Table 2:** The percent amount of vancomycin eluted within 120 h from each specimens

	Vancomycin only	Vancomycin + NaCl	Vancomycin + PVP
Solid	10	4	10
Tube	27	17	32
Large honeycomb	80	52	87

These results demonstrate that PVP enhanced vancomycin elution, particularly in hollow and large-channel honeycomb structures, while NaCl generally had a suppressive effect on drug release.

### Antibacterial Assay Results

**Fig. 3:**
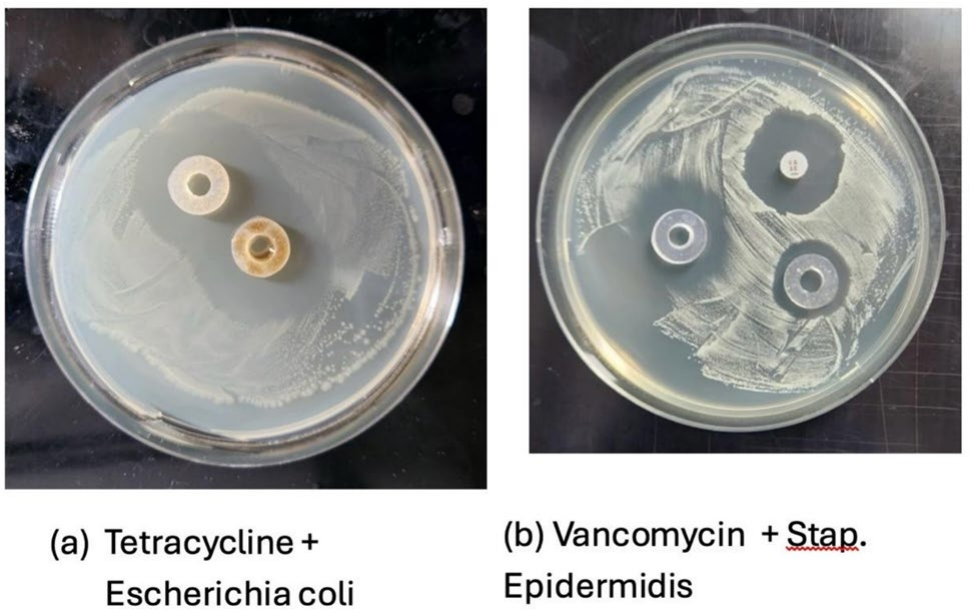
Antibacterial assays

Both tetracycline and vancomycin 3D-printed cylinders demonstrated an acceptable zone of inhibition, consistent with their antibacterial effects (see Fig. 3), suggesting that the efficacy of the antibiotics is not impacted by the 3D printing or manufacturing process.

## Discussion

The findings of this in vitro study demonstrate the potential of 3D-printing technology to fabricate antibiotic-eluting beads with customizable and predictable elution profiles. The honeycomb structures exhibited promising release kinetics and yields, suggesting that 3D-printed antibiotic-loaded beads may be suitable for orthopedic applications, where sustained drug delivery is crucial. The differences in elution profiles between vancomycin and tetracycline underscore the importance of considering the molecular properties of drugs when designing 3D-printed drug delivery systems. We hypothesize that the large molecular structure of vancomycin acted as its own porogen, thereby minimizing the additional benefit of using a larger-structure porogen, such as PVP. Conversely, the porogen had more pronounced effects in the tetracycline samples, achieving the desired macro-porosity and enhancing release characteristics.

Prosthetic joint infections (PJIs) and chronic osteomyelitis pose significant clinical challenges, requiring effective local antibiotic therapy for infection control. While traditional polymethylmethacrylate (PMMA) beads are commonly used, they have several limitations: they are labor-intensive and messy to prepare, expose surgical staff to volatile monomer vapors, and suffer from inconsistent drug distribution due to manual mixing, leading to unpredictable drug release profiles. Commercially available antibiotic cement spacers, such as the Remedy Spacer series (OsteoRemedies, Memphis TN), offer solutions in the PJI and joint reconstruction space, but managing bone loss and abnormal anatomy remains highly technique-dependent and time-consuming intraoperatively. Furthermore, these commercial spacers do not offer varying porosities or elution profiles tailored to maximize effective drug concentrations and minimize the total dose required. To the authors’ knowledge, no pre-formed antibiotic beads are currently available for trauma and bone loss applications. The integration of 3D-printing technology with antibiotic-eluting materials has the potential to overcome these limitations by offering customizable designs with precise control over shape, porosity, and drug release kinetics, paving the way for safer and more effective local antibiotic delivery systems.

The use of 3D-printing technology, particularly resin-based stereolithography (SLA), offers numerous advantages over traditional methods for creating antibiotic-eluting materials. One major benefit is its ability to save operating room (OR) time by providing ready-to-use, “plug-and-play” solutions that eliminate the need for messy, labor-intensive preparation during surgery. In addition, SLA printing reduces health hazards by avoiding exposure to harmful monomer vapors associated with PMMA bead preparation. The technology allows for customizable drug release through precise control over the geometry and porosity of the printed structures, enabling tailored treatment for individual patients. SLA printing is also cost-effective, user-friendly, and capable of preserving the integrity of antibiotics due to its low-temperature processing. With fast print times—typically under 1 h—SLA 3D printing offers a practical and efficient alternative for producing advanced local antibiotic delivery systems.

Timed release is particularly valuable in orthopedic infections, where an initial high concentration of antibiotics can target the early acute phase of infection, followed by a controlled taper to prevent bacterial regrowth and biofilm formation over time. This strategy minimizes the risk of antibiotic resistance by reducing unnecessary exposure while maximizing therapeutic impact precisely when needed most. Moreover, 3D-printed PMMA structures could extend beyond joint infection management to address a broader spectrum of orthopedic infections, enabling personalized treatment that adapts to the specific needs of each patient and infection site.

While this proof-of-concept investigation successfully established feasibility, it is essential to acknowledge the limitations inherent in our experimental design. Specifically, the findings are constrained by a small sample size of one replicate per test group, precluding the robust application of statistical analysis to determine significance. Consequently, future research will involve conducting a multi-sample series to rigorously assess inter-sample variability, identify potential inconsistencies arising from the production process, and facilitate the statistical validation of the observed trends.

## Conclusions

These findings highlight the promising role of 3D-printing technology in developing next-generation antibiotic-eluting implants with tailored release kinetics. Future in vivo studies are warranted to validate these findings in a clinical setting. By leveraging 3D-printing technology and porogen additives, local drug delivery systems can be precisely engineered with optimized internal structures, enabling controlled drug release at any desired rate, ensuring complete drug delivery, and reducing the initial drug amount required—representing a significant advancement in therapeutic efficiency.
